# How AI anxiety and contextual factors shape employee deviant innovation

**DOI:** 10.3389/fpsyg.2026.1906727

**Published:** 2026-08-24

**Authors:** Zheng Ma, Hanwen Lin, Yanshu Ji

**Affiliations:** 1 School of Economics and Management, Huainan Normal University, Huainan, China; 2 Dongfang College, Zhejiang University of Finance and Economics, Haining, China; 3 School of Business, Nanjing University, Nanjing, China

**Keywords:** causal complexity, complex systems thinking, deviant innovation behavior, employee psychology, fuzzy-set qualitative comparative analysis, technology-organization-environment framework

## Abstract

Although employees’ deviant innovative behavior lacks formal authorization, it may become a key driver of organizational innovation performance in the digital era. However, its generation mechanism—shaped by the interplay of psychological perception, organizational design, and external institutional forces—remains underexplored, particularly in the Chinese context. To investigate the motives for innovation in a turbulent era, this paper adopts the Technology-Organization-Environment (TOE) framework as the analytic system and applies fuzzy-set Qualitative Comparative Analysis (fsQCA) to carry out a configurational study of sample data from 326 corporate employees in China. Focusing on six antecedent conditions including AI anxiety, generative AI skills, gamification of platform work, autonomous decision-making, job insecurity and government support. Based on the empirical study, the following have been found: (1) No single element in the system needs to be triggered to motivate employees’ deviant innovation behavior; (2) There are three equivalent system pathways for achieving high levels of employee deviant innovation behavior, categorized as the Anxiety-autonomy driven pathway, the External supported driven pathway, and the Passive triggered driven pathway; (3) A specific combination of conditions inhibits deviant innovation (Low activation environment inhibited type), demonstrating asymmetric causal complexity with the high-drive pathways. This study breaks through the limitations of traditional linear analysis, broadens the application boundaries of the TOE framework in innovation management from a complex systems perspective, and provides valuable theoretical support and practical guidance for systematically stimulating employees’ innovative potential in localized digital contexts.

## 1 Introduction

Against the backdrop of AI’s widespread adoption and increasing market competition, breakthrough innovation can provide strong support for organizations to enhance their competitiveness (Li Z. et al., 2025). Recent evidence suggests that generative AI is continuously reshaping how employees perceive, evaluate, and implement ideas, while workplaces are also being affected, with anxiety and job insecurity gradually intensifying. In the face of complex environments, high-performance work systems and the intrinsic motivation they trigger among employees have a crucial impact on the overall innovation performance of an organization (Wang et al., 2024). Concurrently, examining the underlying psychological triggers of individual proactive behavior provides a foundational lens for understanding how constructive workplace outcomes can be systematically cultivated within rapidly shifting corporate environments (Bai et al., 2022). Only by continuously exploring the triggering mechanisms of employees’ innovative capabilities can enterprises promote breakthrough innovation, create greater value, and ensure stable future development.

As innovative behavior discovered and emerged in recent years, deviant innovation has gradually gained recognition from many scholars, who have conducted research on it from various perspectives. The number of related studies is abundant, leading to a lack of uniformity in the definition of its concept and connotation. The widely accepted academic concept of deviant innovation is: employees engage in innovation activities aimed at improving organizational performance in unauthorized organizational contexts, characterized by informality, concealment, bottom-up nature, and confrontational features, ultimately creating more value for the enterprise. This concept can be divided into two major subtypes: first, bootleg innovation, with concealment as its core characteristic (Augsdorfer, 2005, 2008); second, creative deviance, with confrontation as its core characteristic (Mainemelis, 2010). In this study, we use “deviant innovation behavior” as an overarching term. The covert “bootleg innovation” and confrontational “creative deviance” are distinguished as its subtypes in the research, but both share the core characteristic of being “unauthorized yet beneficial to the organization. “Recent empirical advances further illustrate that non-traditional cognitive dimensions and playful personal traits play an explicit role in activating these self-directed, covert innovative pathways within the modern workforce (Liu et al., 2024). At the same time, with the deepening of digitalization, employees’ awareness and perception of AI are increasingly affecting their paths to innovation and behavior (Li Z. et al., 2025). As the company has carried out digitalization, some employees have started to experience various forms of stress from technology; this may affect their mental health and even inhibit the generation of new ideas (Cantas et al., 2026).

Most existing studies examine individuals, leaders, or organizations in isolation, yet overlook the fact that deviant innovation is inherently a multi-level phenomenon. It originates from individual perception, unfolds within organizational structures, and is shaped by broader external forces. The limitations of a single perspective fail to capture the generative mechanisms of deviant innovative behavior. If the mechanisms of deviant innovation behavior can be explored by integrating the three levels and the reasons for their occurrence identified, this will offer theoretical support and provide reference for enterprises to stimulate employees’ innovative capabilities and continuously enhance their core competitiveness. Deviant innovative behavior does not necessarily stem from a desire for self-interest in the interest of the organization; rather, it can promote the development of an innovative enterprise. An increasing number of researchers are turning their attention to employees’ deviant innovative behavior, hoping to explore its triggering mechanisms in order to improve the overall innovation capacity and performance of organizations (Dahling and Gutworth, 2017).

Existing research holds varying views on deviant innovation. Some scholars define it as a covert, individual-based innovative behavior driven by intrinsic motivation, secretly carried out during the enterprise’s development process. It is typically a bottom-up, unplanned activity that lacks formal authorization from management and is characterized by its hidden nature (Augsdorfer, 2005); Other scholars also believe that deviant innovation refers to behavior that, despite explicit prohibitions from management, is pursued by employees who, after personal deliberation, deem the action worthy of further exploration and begin advancing their ideas in unconventional ways, characterized by a confrontational nature (Mainemelis, 2010). The dual concept of employee deviant innovation has sparked a series of debates: is it a powerful social behavior of organizational commitment, or a risky behavior that threatens managerial authority? Recent researchers suggest that it depends on contextual factors, such as leadership responses (Lin et al., 2016), the organization’s tolerance for rule-breaking, and the perceived legitimacy of innovation goals for organizational benefits (Dahling and Gutworth, 2017). Especially when an organization is in crisis or facing a highly uncertain external environment, high-quality employee relationships and satisfaction can even translate into a key driving force for promoting resilient innovation (Shang et al., 2026).

Existing research implicitly assumes that deviant innovation follows a linear, additive logic. However, this assumption contradicts the theoretical essence of deviant innovation. Deviant innovation behavior arises from the interplay of individual initiative, organizational authorization, and environmental pressure. When factors at different levels are studied in isolation, it is impossible to understand how they interact, substitute, or whether multiple combinations can lead to equivalent outcomes. These questions precisely require a configurational rather than a linear analytical approach. The narrow focus of this structure fails to consider that unauthorized creative behavior has various forms and, depending on the type of leadership, can be either beneficial or harmful. Although many studies have investigated the various links among individuals, leaders and organizations in recent years, few have focused on how changes in technology have affected the causes of deviant innovation. Technology has been driving economic growth and providing full-process support for the all-round development of the country. Research will be carried out on the impact of technical factors and deviant innovation.

To improve the reproducibility of the study, the technical-level influence in the context of deviant innovation is transformed into two measurable dimensions: generative AI competence and AI anxiety, and well-established scales are selected for measurement. Both have met the requirements for reliability and validity, and their effects on deviant innovation will be analyzed using fsQCA analysis. This way can extend the background conditions for the trigger mechanism of deviant innovation and address the current deficiency of local-level research in this area. At the same time, environmental macro-pressures, such as job insecurity, are also important relational stressors that can affect a person’s mind and will to create in innovative activities (Lassleben, 2023). To understand the various types of job-related anxiety, it is necessary to address the problem of resource depletion and uncertainty in action that may result from such anxieties; otherwise, there will be no motivation for proactive behavior (He K. et al., 2022). At the same time, existing research has not established an organized system for exploring its formation reasons. There is a lack of research on how technological conditions, organizational environments and the external environment interact to affect the internal mechanisms of deviant innovation behavior. Thus, few explanations for the many reasons behind this behavior have been put forward, and enterprises are unable to take specific corrective actions.

Tornatzky and Fleischer proposed the TOE (Technology-Organization-Environment) framework to systematically study the interactions among the three levels of technology, organization and environment in the form of a basic analytical tool. As the TOE framework has been applied by scholars in many places over time, its applications have varied and can be used in multiple ways. However, when exploring new research subjects, careful selection of relevant elements is necessary. In research on corporate innovation, some scholars have also applied the TOE analysis framework. For example, some scholars have mentioned in their study the exploration of corporate original innovation based on the TOE framework. The introduction of this perspective is crucial, because sustainable innovation in complex systems is often not the breakthrough of a single node, but the result of the co-evolution and accumulation of multidimensional elements over time (Chu, 2025).

This paper argues that the TOE framework’s analytical approach is also applicable to deviant innovation, which falls within the category of innovation. Qualitative comparative analysis (QCA) is highly suitable for our investigation because the theory of deviant innovation behavior involves multiple different combinations of conditions that can lead to the same outcome. This aligns well with QCA’s principle of equifinality. Different employees may reach deviant innovation behavior through different motivational pathways: some driven by a combination of anxiety and autonomy, while others are driven by external support in the absence of internal authorization. These are precisely the configuration patterns that QCA is designed to identify (Fiss, 2007; Ragin, 2009; Fiss, 2011). Furthermore, QCA has the characteristic of revealing that the conditions under which an outcome occurs are systematically different from those under which it is absent. The inhibiting factors of deviant innovation may not simply be the opposite, which aligns with the theoretical relevance of QCA. In fact, the fsQCA method, grounded in a complex systems perspective, has demonstrated high explanatory power and applicability in academia for deconstructing multi-factor coupling within innovation ecosystems and identifying collaborative innovation pathways (Xu et al., 2026). Therefore, this study, based on the TOE framework, systematically explores multiple driving paths of employees’ deviant innovation behavior from the three dimensions of technology, organization, and environment using the QCA method, and analyzes the synergistic mechanisms under different combinations of conditions. Because in an open system environment, the degree of synergy and coupling among multi-dimensional factors will directly determine whether the ultimate innovation performance can emerge (He K. et al., 2022, He X. et al., 2022). To compensate for the lack of attention in existing research on the combined effects of multiple factors, deepen the understanding of the formation logic of employees’ deviant innovation behavior, and thereby provide theoretical basis and practical reference for enterprises to stimulate employee innovation and guide the orderly development of deviant innovation behavior. Methodologically, we integrated the TOE framework with fsQCA, shifting the analytical perspective from net effects to system effects, thereby generating new insights that neither approach alone could offer. This theory-methodology model allowed us to demonstrate that theoretically “negative” conditions, such as AI anxiety, can become positive drivers of deviant innovation when configured with enabling organizational or environmental factors. This finding challenges the additive causality assumption implicitly held in most prior studies on deviant innovation behavior. This study attempts to answer the following question: the pathways that trigger employees’ deviant innovative behavior, revealing the phenomenon of deviant innovative behavior in China through a localized perspective.

## 2 Theoretical foundation and research model

### 2.1 Theoretical background

#### 2.1.1 The multidimensional evolutionary logic of creative deviance

Knight (1967) was the first to discover deviant innovation during an observation of internal innovation in a company. It refers to the situation in which employees, after generating improvement ideas but lacking formal organizational authorisation, choose to temporarily conceal their activities and bypass management intervention to promote the realization of their ideas in an “underground manner.” Subsequently, two main perspectives have been developed based on this: implicit “bootlegging” and explicit “creative deviance” (Augsdorfer, 2005; Masoudnia and Szwejczewski, 2012). Both are expected to enhance organizational performance and show the employees’ own initiative in the absence of praise or encouragement. Deviant innovation is not necessarily harmful to the interests of the organization; rather, it can enhance the competitiveness of the organization and promote new-area innovation (Dahling and Gutworth, 2017). At present, some scholars have studied deviant innovation behavior based on the Trait Social Activation Theory (Tett and Burnett, 2003) and Social Cognitive Theory (Bandura, 2001). Griffin et al. (1993) put forward the “Organizational Creativity Interaction Model” and also believe that all links at different levels in a large-scale organization are interrelatedly influencing the work of creative employees. However, most of the existing research has only conducted a single-perspective net effect analysis and has failed to study how multiple levels of factors interact or to conduct systematic exploration based on local conditions (Tenzer and Yang, 2019; Liu et al., 2024). Based on the above, this paper will use social cognitive theory and the interaction theory of creativity as its foundation to explore the antecedent complexity of deviant innovation from an all-encompassing perspective.

#### 2.1.2 Applicability of the TOE systematic analysis framework

Tornatzky and Fleischer put forward the Technology-Organization-Environment (TOE) model (DePietro et al., 1990), and many researchers have studied the interdependence of all three over the years. We argue that this framework is particularly suitable for studying deviant innovation behavior for three reasons.

First, although deviant innovation behavior is carried out by individual employees, it indeed captures multi-level logic. When operationalizing the TOE framework for research on deviant innovation behavior, we defined the three dimensions as follows: the technological dimension focuses on employees’ perception and use of AI tools (AI anxiety and generative AI competence); the organizational dimension emphasizes workplace design and leadership mechanisms (platform work gamification and autonomous decision-making); the environmental dimension highlights external threats and institutional buffers (job insecurity and government support). These six conditions collectively interact to influence employees’ decisions on whether to engage in deviant innovation.

Second, compared to alternative frameworks, the TOE framework has clear advantages. Institutional theory emphasizes that organizations adopt similar structures and policies to gain social legitimacy (Amenta and Ramsey, 2010). In the context of deviant innovation behavior, institutional theory would predict that employees and organizations implement external compliance measures to demonstrate fitness. However, it fails to fully reveal organizational micro-mechanisms such as autonomy and gamification, through which employees often experience and respond to external pressures. Sociotechnical systems theory focuses on the interdependence between social and technical subsystems but remains limited to surface-level descriptions and has not yet established complete configurational logic to analyze the coupling effects of multiple conditions (Walker et al., 2008). In contrast, the TOE framework emphasizes how conditions from different dimensions combine, rather than how each single factor independently predicts outcomes. While providing a concise and systematic classification of conditions, it retains configurational logic. This configurational orientation aligns well with our fsQCA methodology.

Third, our application of the TOE theoretical framework involves conceptual adjustments rather than a direct replication of the original framework. Previous studies applying the TOE framework have typically decomposed the technological, organizational, and environmental factors into independent additive predictors for regression analysis. In contrast, this study reconceptualizes them to depict interactive and synergistic complex causal mechanisms. No single dimension operates independently; the causal explanatory power of each dimension is contingent on the mutual alignment of elements from the remaining dimensions. Compared to traditional net effect analysis, this approach places greater emphasis on the configurational effects of multi-factor interaction and synergy, thereby better restoring the original configurational intent of the TOE theory.

### 2.2 Multiple antecedent conditions from a configurational perspective

The selection of the six antecedent variables is based on contextual and theoretical considerations. Under the core premise of the TOE framework, we choose variables related to the technology, organization, and environment dimensions. To ensure the principle of systematic coverage, we identify the factors most closely associated with employees’ deviant innovation behavior from each dimension. That is, factors that employees directly perceive, experience, or are influenced by in their daily work in the current era. To adhere to this principle, in the technology dimension, we select employees’ subjective perceptions of AI tools and their competence; in the organization dimension, we choose the structure and motivation of their direct work environment; and in the environment dimension, we select external pressures and institutional support related to perceived risks and opportunities.

We acknowledge that prior research on deviant innovation behavior has identified other significant predictors, such as leadership style, psychological safety, and organizational support. However, these variables were not included in the study for two reasons. First, they are primarily endogenous to the organization and are better conceptualized as antecedents operating at the organizational or interpersonal level, rather than as independent dimensions. Second, and more importantly, our goal is not to exhaust all possible antecedent variables related to deviant innovation behavior, but rather to test a theoretically parsimonious model that captures cross-dimensional interactions among technology, organization, and environment—interactions that prior research, which largely focused on internal organizational factors, has largely overlooked. Therefore, the exclusion of such variables reflects a deliberate theoretical boundary, rather than an oversight.

#### 2.2.1 Technological dimension: the technological pull of AI anxiety and generative AI competence

At the technical level, AI anxiety refers to employees’ concerns, worries, or misunderstandings about human-machine relationships that arise during the use of artificial intelligence (Li and Huang, 2020; Johnson and Verdicchio, 2017). This cognitive uncertainty and emotional tension may weaken employees’ courage to explore deviant innovation; however, it may also, due to the perceived threat of eroded professional value, inversely motivate employees to prove their self-worth through deviant innovation. In the consideration of whether AI anxiety may suppress or stimulate deviant innovation behavior, a key question arises: under what conditions do each effect dominate? We propose that the answer lies in cognitive appraisal. If employees perceive abundant organizational resources such as autonomous decision-making power, platform gamification incentives, and external policy safeguards, they will interpret AI anxiety as a challenge signal (Li H. et al., 2025), generating growth-oriented approach motivation, thereby promoting deviant innovation. Once supporting resources are scarce, the same anxiety perception will be judged as a threat signal, inducing defensive withdrawal and behavioral inertia. We acknowledge that classifying AI anxiety as a technical factor may seem counterintuitive, as anxiety is essentially a psychological state. However, our classification primarily reflects the distinction between the object of anxiety and the source of anxiety. In this study, AI anxiety is not a general anxiety trait but rather the perception and response to the context of technological change. We consider it as an individual perception of technology and capturing this evaluation dimension places it within the TOE technology dimension. Meanwhile, generative AI competency also plays a key role. Generative artificial intelligence can significantly enhance employees’ innovation and performance (Li et al., 2024). However, its impact on deviant innovation is conditional rather than direct. Employees can use generative AI to quickly gain inspiration and validate hypotheses, thereby exploring informal innovation solutions at a lower cost and reducing the psychological uncertainty associated with “rule-breaking” and failure. However, such enabling effects cannot take effect independently and may only manifest under two types of configuration conditions: first, employees have sufficient organizational autonomy to implement AI-generated innovative suggestions; second, an external support system can buffer the adverse effects of trial-and-error failures.

#### 2.2.2 Organizational dimension: contextual stimulation of platform work gamification and autonomous decision-making

At the organizational level, platform work gamification refers to the application of game elements in non-game environments as a special persuasive mechanism to enhance employee engagement and alter behavioral motivation (Hamari and Koivisto, 2015a,b; Hamari, 2014; Sharp and Sharp, 1997). Gamification design has a dual impact on deviant innovation: on one hand, the “safe failure” environment it creates can provide employees with a sense of autonomy and positive motivation, reducing the perceived psychological risk of deviant behavior; On the other hand, if gamification is highly tied to performance monitoring, it will exert the constraining function of organizational control, increasing the risk of deviant innovation being “identified” (Hamari et al., 2015). Additionally, autonomous decision-making empowers employees with the flexibility to respond without managerial intervention (Gonzalez-Mulé et al., 2016). When employees possess sufficient knowledge but lack formal authorization, institutional constraints may drive them to seek informal pathways; conversely, a leader’s timely delegation of authority can effectively unlock their potential for deviant innovation.

#### 2.2.3 Environmental dimension: external push of government support and job insecurity

At the environmental level, job insecurity stems from uncertainty about job continuity or the risk of job displacement caused by AI (Mauno et al., 2001; Greenhalgh, 1984). This sense of unease may motivate employees to try high-return innovative approaches beyond existing paths in order to cut losses or prove themselves; However, it may also lead to employees sticking to the rules and suppressing deviant behavior due to being overly sensitive to the risk of organizational punishment. To clarify the classification logic, this study uses the source of anxiety as the basis for categorization, rather than the specific object that triggers anxiety. Regarding the classification ambiguity between AI anxiety and job insecurity, this study posits that AI anxiety arises from employees’ interactive experiences with AI, representing a type of technology evaluation response. In contrast, job insecurity originates from individuals’ perceived risk of labor market fluctuations, with their triggers not limited to AI-related concerns about job continuity and career prospects but rather falling within the realm of external labor market structures. Therefore, this study categorizes AI anxiety under the technology dimension, while job insecurity is classified under the environmental dimension. This aligns with prior research that views job insecurity as a perception of environmental stressors rather than technology-specific pressures (Mauno et al., 2001; He K. et al., 2022). At the same time, in the context of China’s transitional economy, the government provides essential external resources and signals of legitimacy. This paper proposes that government support drives employees’ deviant innovation behavior through three interrelated pathways. The first is the regulatory legitimacy mechanism: government-issued supportive policies and special funds send clear signals, confirming that relevant innovative activities are socially recognized, thereby weakening employees’ perception of the risk of organizational punishment for non-compliant innovation. The second is the normative legitimacy mechanism: the government’s strong promotion of AI implementation and innovation development shapes normative consensus at the industry and societal levels, encouraging employees to proactively experiment with new technologies and innovative solutions. The third is the resource buffering mechanism: tangible resources such as government subsidies, tax reductions, and specialized talent support can share the costs of innovation trial and error, significantly reducing the personal losses and career development risks employees bear when engaging in deviant innovation. In summary, government support can positively legitimize and empower deviant innovation by reshaping employees’ perceptions of acceptable and feasible behaviors within the organizational context, while providing enabling support for innovative practices.

### 2.3 Construction of a theoretical framework for multi-factor concurrence

**Figure 1:**
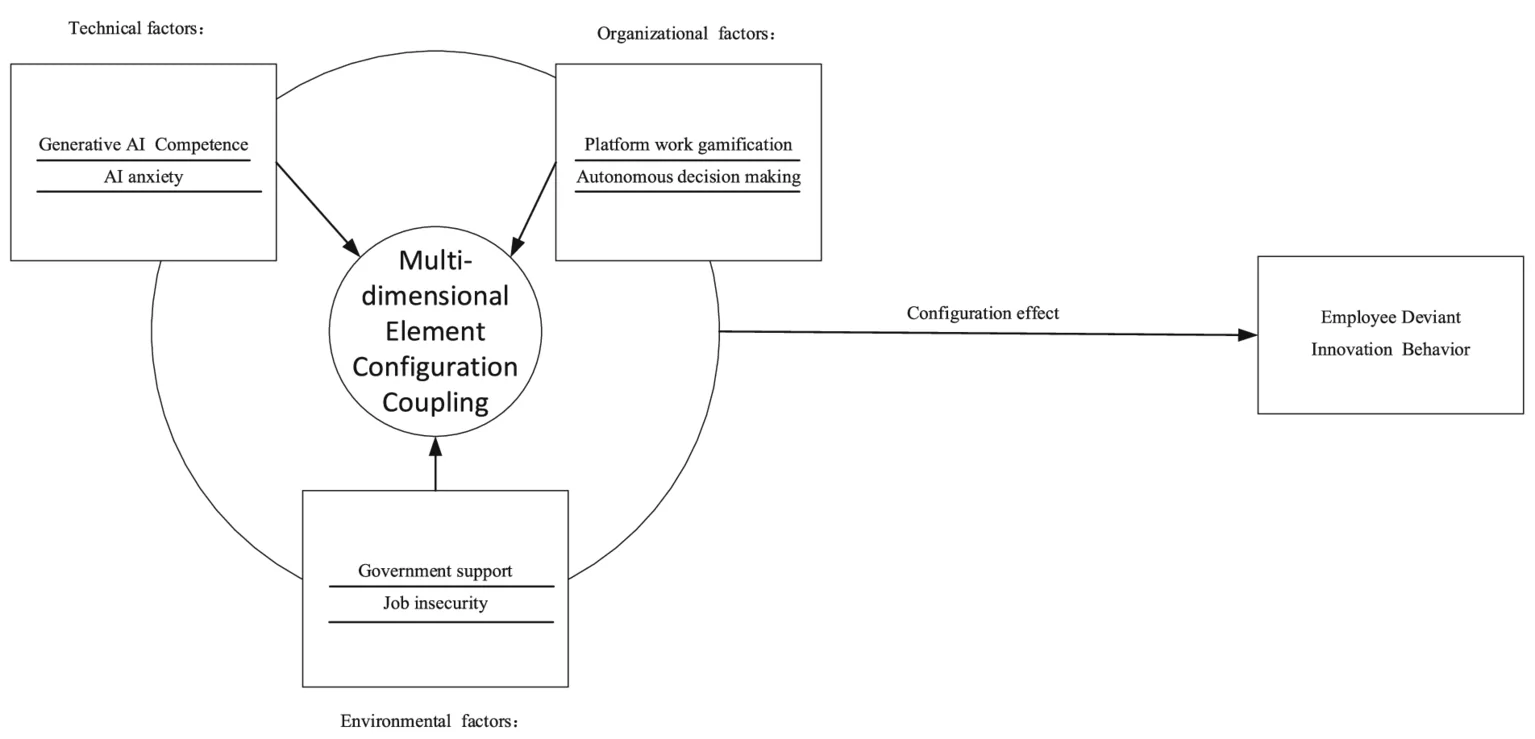
Research model.

From the perspective of complex systems and configuration, the six antecedent variables in the three dimensions of technology, organization, and environment do not influence employees’ deviant innovation behavior independently; instead, they work together through linkage and matching. Therefore, based on the TOE framework, this paper delves into how the three groups of factors—technology, organization, and environment—along with their six antecedent variables, interact and synergize to ultimately influence the emergence of employees’ deviant innovation behavior, thereby constructing the theoretical analysis framework of this paper, as shown in Figure 1.

## 3 Research design

### 3.1 Research method

This paper adopts the qualitative comparative analysis (QCA) method proposed by Ragin (2000), which is a set-theoretic approach aimed at exploring the influence of configurations composed of multiple factors on a particular outcome. The QCA method was adopted mainly for the following two reasons: First, from a configuration perspective, the six antecedent conditions within technological, organizational, and environmental factors are not independent of each other but interact through a linked and matching approach. Second, there are multiple combination pathways among the various triggers of employees’ deviant innovation behavior, but it remains unknown how exactly these factors should be combined to achieve the desired outcome. The selection of QCA’s two methodological foundations in this paper is not random but derived from the core internal logic of the TOE framework. TOE emphasizes the interdependence of technological, organizational, and environmental dimensions, rejecting traditional additive variable thinking and positing that the role of elements relies on cross-dimensional interactions rather than independent net effects. Based on this theoretical premise, two expectations suitable for configurational analysis can be derived: first, TOE elements function together as combined configurations, and the unit of analysis should be sets of conditions rather than isolated variables; second, cross-dimensional interactions exhibit equifinality, where different element configurations can compensate for one another and lead to the same outcome. Therefore, the application of QCA is not merely a tool-level choice but a logical extension of the TOE framework theory, completing the empirical operationalization of the theoretical configurational thinking. Therefore, this paper takes the technological factors of generative AI competence and AI anxiety, the organizational factors of platform work gamification and autonomous decision-making, and the environmental factors of government support and job insecurity as antecedent conditions. The antecedent and outcome variables of the study were measured using established scales from both domestic and international sources, with a seven-point Likert scale. The data was collected through a questionnaire targeting corporate employees, yielding a total of 326 valid samples. The sample covered different regions, enterprise types, and job levels. This study uses fsQCA 3.0 software for variable calibration, necessity analysis, sufficiency configuration analysis, and robustness analysis to systematically explore the causal complex mechanisms of employees’ deviant innovative behavior. Since all variables were measured via self-report questionnaires at a single time point, common method bias may pose a threat to the validity of the research conclusions. Although the fsQCA method is less affected by common method bias, we still implement both procedural and statistical preventive measures. Procedurally, we ensured respondent anonymity, selected relatively mature scales for measurement, emphasized that there were no right or wrong answers, and used reverse-scored items to reduce response consistency bias. Statistically, Harman’s single-factor test was conducted on all measurement items. The results showed that the first factor accounted for 27.6% of the variance, well below the critical threshold of 40% (Podsakoff et al., 2003), indicating that the data in this study do not suffer from severe common method bias.

### 3.2 Sample survey

The data in this paper is derived from a questionnaire survey. The research subjects are primarily employees of enterprises in various regions, and data was collected through the questionnaire survey, with a total of 326 valid questionnaires retrieved. Among the surveyed employees, males account for 49.08% and females account for 50.92%; The proportion of those under 30 years old is 26.07%, those aged 30–45 (inclusive) account for 50.61%, those aged 45–55 (inclusive) account for 19.33%, and those over 55 account for 3.99%; The proportion of those with an education level of junior college or below is 42.94%, bachelor’s degree accounts for 49.39%, master’s degree accounts for 6.75%, and doctorate or above accounts for 0.92%; In terms of enterprise nature, employees of state-owned enterprises account for 28.22%, private enterprises 45.40%, foreign-funded enterprises 10.74%, collective enterprises 9.51%, and other types of enterprises 6.13%; In terms of positions, grassroots employees account for 74.85%, grassroots managers account for 18.40%, and middle and senior managers account for 6.75%; In terms of enterprise size, employees in companies with 50 or fewer people account for 20.86%, those in companies with 51–300 employees account for 36.20%, those in companies with 301–500 employees account for 15.34%, and those in companies with 500 or more employees account for 27.60%; Employees in companies aged under 5 years account for 14.42%, those in companies aged 5–10 years (inclusive) account for 19.63%, those in companies aged 10–30 years (inclusive) account for 48.47%, and those in companies aged over 30 years account for 17.48%; The proportion of those with less than 1 year of work experience is 14.11%, those with 1–5 years (inclusive) account for 35.28%, those with 5–10 years (inclusive) account for 28.83%, and those with more than 10 years account for 21.78%; Employees in the Eastern region account for 33.44%, the Central region for 30.98%, the Western region for 22.09%, and the Northeastern region for 13.49%.

### 3.3 Variable measurement

The results of this study include deviant innovation behavior, antecedent conditions such as AI anxiety, generative AI competence, platform work gamification, autonomous decision making, job insecurity, and government support. All items in the survey questionnaire use a 7-point Likert scale (1 = strongly disagree, 7 = strongly agree), To ensure the reliability and validity of the scale, existing mature scales from both domestic and international sources were adopted. For scales originating from foreign studies, the researchers first translated them into Chinese and conducted a pre-survey before distributing the formal questionnaire. Based on feedback and suggestions, the scales were appropriately modified to better fit the research context, resulting in the final survey questionnaire for this study.

Generative AI Competence. The generative AI competence scale developed by Lee et al. (2025) was adopted, consisting of 9 items. Typical items include, “I can proficiently use generative AI to complete work tasks” and “I can evaluate the accuracy of outputs generated by generative AI.” The Cronbach’s *α* coefficient of this scale in this paper is 0.945.

AI anxiety. Referring to the AI anxiety dimension scale developed by Li et al. (2022), this scale consists of 6 items. Typical items include “I worry that AI will replace part of my work” and “The rapid development of AI technology makes me feel uneasy.” The Cronbach’s *α* coefficient of this scale in this paper is 0.915.

Platform work gamification. Referring to the study by Hamari and Koivisto (2015a,b), this scale comprises 11 items. Typical items include “The gamification design of the platform makes work more interesting” and “The virtual rewards obtained from completing tasks enhance my work motivation.” The Cronbach’s *α* coefficient of this scale in this paper is 0.948.

Autonomous decision-making. Using the autonomous decision-making measurement scale developed by Gonzalez-Mulé et al. (2016), this scale consists of 4 items. Typical items include “I can independently decide how to complete my work” and “I have a significant say in the arrangement of work tasks.” Cronbach’s *α* coefficient of this scale in this paper is 0.877.

Job insecurity. Using the job insecurity scale developed by Mauno et al. (2001), this scale comprises 5 items. Typical items include “I worry about losing my current job in the future” and “The instability of the company makes me anxious about my career prospects. “The Cronbach’s *α* coefficient of this scale in this paper is 0.882.

Government support. Based on the study by Wang and Sun (2023), this scale consists of 6 items, with typical items such as “The government provides policies and projects that are beneficial to the development of my company” and “The government offers significant assistance for my company to obtain financial support.” The Cronbach’s α coefficient of this scale in this paper is 0.907.

Deviant innovation behavior. Based on the study by Lin et al. (2016), this scale consists of 9 items, with typical items such as “Even if not formally authorized, I try to improve work processes” and “To achieve more efficient solutions, I break through existing rule constraints.” The Cronbach’s α coefficient of this scale in this paper is 0.944.

### 3.4 Reliability and validity analysis

The results of the new validity test for the questionnaire data collected are shown in Table 1. By using SPSS 31.0 software to calculate the Cronbach’s α values of each scale to test reliability, the Cronbach’s α values of the seven scales were all greater than 0.8, indicating that the reliability of each scale is good and acceptable. In terms of validity, the convergent validity of the scales was analyzed through factor loadings, average variance extracted (AVE), and composite reliability (CR). The analysis results show that the factor loadings for the items corresponding to the scales of generative AI competency, AI anxiety, platform work gamification, autonomous decision-making, job insecurity, government support, and deviant innovation behavior are all above 0.6, indicating that each item has high representativeness. Meanwhile, the AVE of each scale is greater than 0.6, and the CR is greater than 0.9, indicating that the scales used in this study have ideal convergent validity.

**Table 1:** Reliability and validity test.

Variable	Item	Cronbach’s α	Factor loading	AVE	CR
AI anxiety	Q1	0.915	0.858	0.703	0.934
	Q2		0.815		
	Q3		0.858		
	Q4		0.861		
	Q5		0.835		
	Q6		0.802		
Autonomous decision-making	Q7	0.877	0.825	0.731	0.916
	Q8		0.861		
	Q9		0.875		
	Q10		0.857		
Platform work gamification	Q11	0.948	0.842	0.661	0.955
	Q12		0.775		
	Q13		0.799		
	Q14		0.777		
	Q15		0.789		
	Q16		0.845		
	Q17		0.796		
	Q18		0.855		
	Q19		0.845		
	Q20		0.850		
	Q21		0.765		
Generative AI competence	Q22	0.945	0.798	0.693	0.953
	Q23		0.813		
	Q24		0.818		
	Q25		0.844		
	Q26		0.790		
	Q27		0.854		
	Q28		0.854		
	Q29		0.829		
	Q30		0.890		
Job insecurity	Q31	0.882	0.873	0.682	0.914
	Q32		0.824		
	Q33		0.798		
	Q34		0.789		
	Q35		0.841		
Deviant innovation behavior	Q36	0.944	0.849	0.691	0.953
	Q37		0.854		
	Q38		0.848		
	Q39		0.782		
	Q40		0.789		
	Q41		0.850		
	Q42		0.875		
	Q43		0.818		
	Q44		0.809		
Government support	Q45	0.907	0.860	0.684	0.928
	Q46		0.804		
	Q47		0.768		
	Q48		0.848		
	Q49		0.824		
	Q50		0.854		

## 4 Data analysis and results

### 4.1 Variable calibration

Before conducting necessity and sufficiency analyzes, data calibration is required (Ragin, 2009; Greckhamer and Gur, 2021). The process of determining the degree of membership of a case in a set based on theoretical knowledge and experience is called calibration (Ragin, 2009), and calibration is one of the key steps in fsQCA. Ragin (2000) proposed three calibration methods: direct assignment, direct calibration, and indirect calibration. Since most of the data samples deviate from 3 after processing the survey data in this study, the scale values cannot be directly used as calibration points. Subsequently, this study draws on existing research by averaging continuous variables such as AI anxiety, generative AI competence, autonomous decision-making, platform work gamification, government support, and job insecurity. Since all variables were measured using a 7-point Likert scale, we considered the scale midpoint as the conceptual crossover point to facilitate the distinction between high and low levels of each construct overall. However, we found that the actual sample data distribution deviated from the scale midpoint. If the scale midpoint were still used as the crossover point, it would result in skewed membership scores that fail to accurately reflect the actual sample. Following established fsQCA practices (Fiss, 2011), we set the 95th, 50th, and 5th percentiles as the calibration points for “full membership,” “crossover point,” and “full non-membership,” respectively. This ensures that membership scores accurately represent the relative positions of respondents within the empirical sample: a method widely used in fsQCA research. This study treats the scale midpoint (4) as a theoretical benchmark for high and low levels of the construct and as a conceptual boundary for distinguishing between high and low states of the variable but does not mechanically use it as an empirical crossover point. Because the sample distribution deviates from the midpoint, arbitrarily using 4 as the crossover point would produce arbitrary membership scores that fail to accurately reflect the relative levels of the respondents. Instead, we use distribution-based anchors to maintain discriminability, a procedure consistent with Ragin’s (2009) recommendation that calibration should reflect empirical distribution while preserving the conceptual foundation. Thus, there is no contradiction. The study uses the scale midpoint for conceptual orientation and percentiles for empirical precision, given the limited variation in the original scale. This preprocessing produced calibration anchors that extend beyond the original 1–7 Likert scale range. Therefore, the study standardized the “autonomous decision-making” variable (mean-centered and divided by the standard deviation) before calibration to enhance cross-construct comparability. In addition, to avoid theoretical difficulties in subsequent research, this paper draws on Fiss’s theoretical research experience and uniformly adds 0.001 to the calibrated data where the value is 0.50, adjusting it to 0.501 (Fiss, 2011). The specific calibration anchors for conditions and outcomes are shown in Table 2.

**Table 2:** Variable anchor selection.

Condition	Variable	Full membership (95%)	Intersection (50%)	Complete non-subordination (5%)
Outcome conditions	Deviant innovation behavior	4.78	3.39	1.89
Antecedent conditions	AI anxiety	5	3.5	2
	Autonomous decision-making.	1.1875	−0.25	−1.75
	Platform work gamification.	4.63	3.18	2
	Generative AI Competence.	5	3.44	2
	Job insecurity	4.6	3	1.6
	Government support	4.83	3.17	1.83

### 4.2 Necessity analysis

In the process of data analysis, the first step is to conduct a necessity analysis, followed by a configuration analysis. Finally, based on the data, it is determined whether the antecedent variable of the study is a necessary condition for the outcome variable of the study. If the condition and outcome are both present or both absent, then the variable can be considered a necessary condition for the outcome variable (Rihoux and Ragin, 2009). As a relevant criterion for measuring necessary conditions, if the consistency of the antecedent condition reaches 0.9 or above, the variable can be judged as a necessary condition for the outcome variable (Schneider and Wagemann, 2012). This study uses fsQCA 3.0 software to conduct necessary condition analysis for high employee deviant innovation behavior and low employee deviant innovation behavior, respectively. The results are shown in Table 3. The test results show that the consistency levels of the single conditions for high employee deviant innovation behavior and low employee deviant innovation behavior are both below 0.9, indicating that there are no necessary conditions for forming high or low employee deviant innovation behavior among the antecedent conditions.

**Table 3:** Necessity analysis.

Antecedent conditions	High deviant innovation behavior		Low deviant innovation behavior	
	Consistency	Coverage	Consistency	Coverage
AI anxiety	0.698709	0.713879	0.590702	0.606273
~AI anxiety	0.61464	0.599181	0.721228	0.706287
Autonomous decision-making	0.702773	0.687032	0.61709	0.606013
~ Autonomous decision-making	0.596987	0.608154	0.681312	0.697215
Platform work gamification	0.713909	0.712988	0.571812	0.573674
~ Platform work gamification	0.573123	0.571261	0.713919	0.714838
Generative AI competence	0.713398	0.691467	0.620481	0.604143
~ Generative AI competence	0.591588	0.608108	0.683124	0.705395
Job insecurity	0.721158	0.7174	0.594479	0.594071
~ Job insecurity	0.591945	0.592353	0.717206	0.720966
Government support	0.701303	0.694513	0.620842	0.617628
~ Government support	0.61389	0.617116	0.692924	0.699735

### 4.3 Sufficiency analysis

This study sets the consistency threshold at 0.8, following the approach of Fiss (2011). Regarding the frequency threshold, it is flexibly determined based on the sample size. Either 1 or a larger value can be used. In this paper, the frequency threshold is ultimately set at 1.5% of the total number of cases, meaning the threshold is 3. Additionally, based on previous research, the PRI consistency was assigned a value of 0.7 (Ragin, C. C., 2009). Using fsQCA 3.0 software to analyze the truth table, through the analysis of complex, intermediate, and parsimonious solutions: conditions that appear in both the parsimonious and intermediate solutions are considered core conditions, while conditions that appear only in the intermediate solution are considered peripheral conditions (Schneider and Wagemann, 2013; Rihoux and Ragin, 2009). The specific analysis is shown in Table 4. There are three realization paths for high employees’ deviant innovative behavior and one realization path for low employees’ deviant innovative behavior. In addition, the consistency of H1a (0.90), H1b (0.90), H1c (0.92), and NH1 (0.88) all exceed the acceptable threshold of 0.8. Moreover, by observing the raw coverage, all four paths can explain a considerable portion of employees’ deviant innovation outcomes.

**Table 4:** Configurational analysis of antecedent conditions for employee deviant innovation behavior.

Conditions	High deviant innovation behavior			Low deviant innovation behavior
	Anxiety-autonomy driven type	Externally supported driven type	Passively triggered driven type	Low activation environment inhibited type
	H1a	H1b	H1c	NH1
AI anxiety	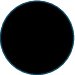	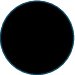		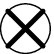
Autonomous decision-making	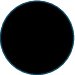		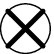	
Platform work gamification	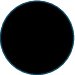	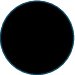	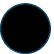	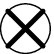
Generative AI competence		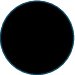	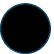	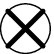
Job insecurity	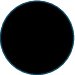	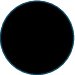	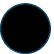	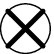
Government support		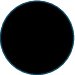	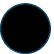	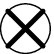
Raw coverage	0.433942	0.384621	0.284499	0.372639
Unique coverage	0.085544	0.00374472	0.0181947	0.372639
Consistency	0.903924	0.915398	0.903178	0.884777
Solution consistency	0.89488			0.884777
Solution coverage	0.470166			0.372639

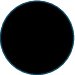
 Indicates the core condition; 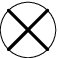 Indicates missing core condition; 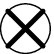 Indicates missing edge condition; 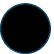 Indicates edge conditions.

### 4.4 Antecedent configuration analysis of high employee deviant innovation

Through horizontal analysis of the configurations in the table, the study found that H1a, H1b, and H1c can all generate high levels of employee deviant innovation behavior, but the driving mechanisms among the three differ. The core conditions of H1a are high AI anxiety, high autonomous decision-making, high platform work gamification, high generative AI competence, and high job insecurity; the core conditions of H1b are high AI anxiety, high platform work gamification, high generative AI competence, high job insecurity, and high government support. H1c exhibits characteristics dominated by marginal conditions, lacking autonomous decision-making, yet featuring a combination of marginal conditions such as platform work gamification, generative AI competency, job insecurity, and government support. This indicates that the realization paths of high-deviation innovative behavior have the characteristic of “different paths leading to the same destination.”

Configuration H1a shows that regardless of generative AI competence and government support levels, the synergistic effect of high AI anxiety, high autonomous decision-making, high platform work gamification, and high job insecurity can stimulate employees’ deviant innovation behavior. It is noteworthy that this pattern holds true across varying levels of generative AI competence and government support. From a configurational perspective, this path reveals a new pattern: the combination of unease about technological cognition with work autonomy, playful engagement, and external employment threats collectively distinguishes high-outcome cases from others.

Configuration H1b indicates that, in the absence of autonomous decision-making conditions, a combination of high AI anxiety, high platform work gamification, high generative AI competence, high job insecurity, and high government support can also drive high deviant innovation behavior. In terms of experience, this configuration reveals a new pattern: the coexistence of external institutional buffering with technical capability, along with high pressure and gamified participation, which compensates for the shortcomings of insufficient internal procedural space. It should be noted that the unique coverage of H1b is extremely low (0.0037), reflecting a high degree of overlap between this path and H1a and H1c, and its explanatory power is relatively weak when separated from the other two configurations. Although the empirical results support the validity of H1b, caution is still needed when generalizing it as an independent “external support” path for theoretical interpretation.

Configuration H1c presents an edge-condition-driven pathway where, in the absence of autonomous decision-making, platform work gamification, generative AI competence, job insecurity, and government support act together as edge conditions to still generate high levels of deviant innovation behavior. In terms of experience, this configuration indicates that despite a lack of autonomy, the synergy between external support, work pressure, and gamification incentives can still differentiate cases of high deviant innovation behavior from other cases.

In summary, the three paths can be categorized as the “Anxiety-autonomy driven type” (H1a), the “External supported driven type” (H1b), and the “Passive triggered driven type” (H1c).

#### 4.4.1 Antecedent configuration analysis of low deviant innovation behavior

Following the causal asymmetry assumption of the QCA method, this paper further analyzes the configurational paths leading to low deviant innovation behavior. The table shows that there is a driving path for low deviant innovation behavior, NH1. The core characteristics of this configuration are absence of AI anxiety, absence of platform work gamification, absence of generative AI competence, absence of job insecurity, and absence of government support, with autonomous decision-making being an irrelevant condition. It indicates that when employees are in this situation, even if they have a certain degree of autonomous decision-making ability, it cannot stimulate deviant innovative behavior. This result further validates the asymmetric causal relationship between high deviant innovation behavior and low deviant innovation behavior.

### 4.5 Robustness analysis

Based on the analytical experience of previous researchers (Schneider and Wagemann, 2012), this paper conducts a robustness analysis by adjusting the case frequency threshold and consistency threshold. First, this paper adjusts the case frequency threshold to 3 and finds that the high employee deviant innovation path remains unchanged. The specific results are shown in Table 5. Secondly, the consistency level was adjusted to 0.85, and it was found that the path influencing employees’ deviant innovation was reduced by only one compared to before the threshold change. When the consistency level was further adjusted to 0.9, the path remained consistent with that at 0.85. The specific results are shown in Table 6. The configuration after changing the relevant thresholds is essentially consistent with that before the change, indicating that the conclusions of this study are robust. In addition to adjusting case frequency and consistency thresholds, we conducted a sensitivity analysis to evaluate whether the core configurations were sensitive to alternative calibration anchors. We recalibrated all variables using alternative anchors (i.e., the 90th, 50th, and 10th percentiles) and reran the fsQCA analysis, with the core configurations remaining largely unchanged.

**Table 5:** Robustness test for increasing case frequency threshold.

Conditions	High deviant innovation behavior			Low deviant innovation behavior
	Anxiety-autonomy driven type	Externally supported driven type	Passively triggered driven type	Low activation environment inhibited type
	H1a	H1b	H1c	NH1
AI anxiety	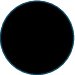	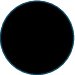		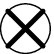
Autonomous decision-making	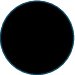		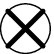	
Platform work gamification	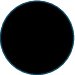	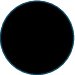	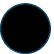	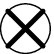
Generative AI competence		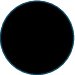	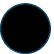	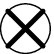
Job insecurity	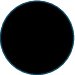	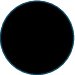	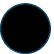	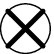
Government support		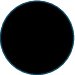	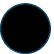	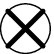
Raw coverage	0.433942	0.384621	0.284499	0.372639
Unique coverage	0.085544	0.00374472	0.0181947	0.372639
Consistency	0.903924	0.915398	0.903178	0.884777
Solution consistency	0.881948			0.884777
Solution coverage	0.48836			0.372639

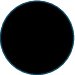
 Indicates the core condition; 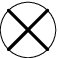 Indicates missing core condition; 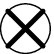 Indicates missing edge condition; 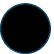 Indicates edge conditions.

**Table 6:** Robustness analysis of increasing the consistency threshold.

Conditions	High deviant innovation behavior		Low deviant innovation behavior
	Anxiety-autonomy driven type	Externally supported driven type	Low activation environment inhibited type
	H1a	H1b	NH1
AI anxiety	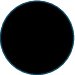	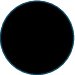	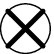
Autonomous decision-making	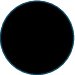		
Platform work gamification	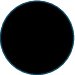	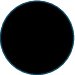	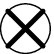
Generative AI competence		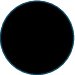	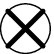
Job insecurity	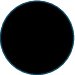	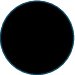	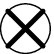
Government support		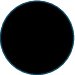	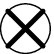
Raw coverage	0.433942	0.384621	0.372639
Unique coverage	0.085544	0.0362234	0.372639
Consistency	0.903924	0.915398	0.884777
Solution consistency	0.89488		0.884777
Solution coverage	0.470166		0.372639

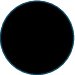
 Indicates the core condition; 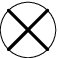 Indicates missing core condition; 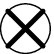 Indicates missing edge condition; 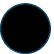 Indicates edge conditions.

## 5 Conclusion and outlook

### 5.1 Research conclusions

Before explaining the configuration findings of the study, it is necessary to clarify the evidentiary boundaries of fsQCA. The fsQCA method identifies which combinations of conditions are sufficient for the presence or absence of an outcome (Schneider and Wagemann, 2012). In this paper, when we describe configurations as driving pathways for deviant innovative behavior, we are more often referring to patterns observed based on empirical reality rather than directly measured causal chains. The psychological explanations we discuss are theories derived from currently observed configuration patterns, rather than direct tests of psychological mechanisms. We make it clear that configurational associations are not absolute assertions, and the theoretical explanations within them require further verification through future empirical practice.

Based on social cognitive theory and the interactionist theory of creativity, this study focuses on the psychological and behavioral mechanisms of employees who engage in self-directed, covert innovation across organizational boundaries. By adopting configurational thinking and utilizing fuzzy-set Qualitative Comparative Analysis (fsQCA 3.0), this paper integrates six core antecedent conditions across technological, organizational, and environmental dimensions. Based on empirical data collected from 326 valid corporate employee questionnaires in China, this study deconstructs the multi-factor concurrent pathways and causal complexity that trigger or inhibit employee deviant innovation behavior. The specific research conclusions are outlined below.

(1) The driving mechanisms of employee deviant innovation are highly non-linear, and no single systemic element constitutes a “necessary condition.”

The necessity analysis reveals that the consistency levels for all six individual antecedent conditions—AI anxiety, generative AI competence, platform work gamification, autonomous decision-making, job insecurity, and government support—fall below the critical threshold of 0.90, whether in their present or absent states. Therefore, employee deviant innovation behavior is not caused by a single psychological stressor or a particular situation in a linear fashion. None of the system-level reasons are required for either the occurrence or absence of this behavior. Instead, deviant innovation is the result of the synergistic effect among multiple conditions, and thus, a configurational approach needs to be taken to decipher non-linear behavior changes in complex digital workplaces.

(2) The generative pathways for high employee deviant innovation exhibit equifinality, characterized by situational dependence and motivational redirection.

The analysis of the sufficiency configuration identifies three equivalent system paths (H1a, H1b, and H1c) that successfully induce high levels of employee deviant innovation behavior. Given that the consistency levels are well above the upper limit of 0.80 and have reached or exceeded 0.90, these routes demonstrate the ways in which modern workers have responded to changes in technology across all areas of motivation:

Anxiety-Autonomy Driven Pathway (H1a): A psychological defense and behavioral redirection mechanism that arises under the stress of technology. When employees feel intense psychological pressure due to continuous AI development (high AI anxiety) and significant job threat (high job insecurity), a strong internal structural support system, such as an autonomous decision-making authority and an appealing platform gamification design, can be introduced to help them manage these anxieties safely. Given the circumstances, even if the technical AI skills of employees and external policy support are only moderate, they will still be motivated to ignore the formal procedures. They actively participate in deviant innovation to demonstrate their own professional value and turn career anxiety into the impetus for innovation.

External Supported Driven Pathway (H1b): This design shows that when internal organizational authorisation is limited, a “psychological safety buffer” is provided by external legitimacy resources. In rigid or conservative organizations that do not grant much space for autonomous decision-making by high-skilled generative AI employees, the pressure of high AI anxiety and job insecurity may be reduced. Through the support of external institutional safeguards, such as strong government support policies, and by embedding them in micro-level platform gamification incentives, these efforts can safely use surrounding resources to implement unsanctioned creative ideas.

Passive Triggered Driven Pathway (H1c): Peripheral elements can work together to create a combined push-pull effect and thus trigger non-formal innovation. Although the company is not fully autonomous, it will provide some marginal incentives to motivate staff better. When a digitized work platform provides gamified intrinsic incentives and employees have a basic level of AI application ability, these internal pulled motives are in opposition to environmental pressures, such as the push of job insecurity and the pull of a structured government support system’s resource. This peripheral synergy can motivate employees to innovate independently by default.

A key clarification is needed here: among the three configuration paths, the unique coverage corresponding to H1b is only 0.0037, indicating that this path adds almost no additional independent explanatory value compared to H1a and H1c. Although the internal logic of “external support replacing autonomy” is theoretically plausible, this configuration can only be regarded as a preliminary inference requiring further testing and should not be considered a stable and reliable empirical conclusion.

(3) The inhibition mechanism exhibits causal asymmetry, where a “low activation environment” creates a deep psychological barrier to innovation.

Assuming the set-theoretic property of causal asymmetry, the configurations that lead to low deviant innovation behavior are not simply the exact opposites of those that cause high deviant innovation behavior. A deficiency analysis has found a single pathway for suppressing deviant innovation, known as the Low Activation Environment Inhibited Type (NH1). This configuration is a completely “blunted” workspace without any of the following: AI anxiety, platform gamification incentives, generative AI literacy, job insecurity, and government support. In the absence of a favourable environment, even with some autonomy in making decisions under the principle of objectivity for employees, their intrinsic motivation will not be fully stimulated. As a result, the employees are still passive and conformist; therefore, psychological activation and behavioral inhibition of workplace innovation operate entirely separate causal logics.

### 5.2 Theoretical contributions

Methodologically, the integration of the TOE framework with fsQCA is not a mere combinatorial operation; it generates unique analytical contributions that neither approach could provide alone. fsQCA facilitates the identification of equivalent causal pathways in research, yet it lacks an inherent theoretical structure for selecting and organizing conditions, whereas TOE offers a more comprehensive classification of conditions. Unlike traditional TOE-based studies, which typically employ regression analysis assuming additive and symmetrical effects, we combine the theoretical comprehensiveness of TOE with the analytical precision of fsQCA to produce new knowledge that cannot be reduced to any individual component. We demonstrate that “negative” conditions, such as AI anxiety, also serve as driving factors in configurations, providing empirical support for the theoretical distinction that the same stressor can be appraised as a threat, leading individuals to either withdraw or engage in challenging behaviors to cope and grow (Biggs et al., 2017). Shifting from analyzing net effects to systemic effects is a departure from prior research, which has rarely addressed this perspective.

Based on the Technology-Organization-Environment (TOE) theoretical framework, this paper builds an integrated framework consisting of technological stress, organizational governance, and environmental pushes to study the triggering mechanisms of employee deviant innovation behavior systematically and deepen the all-round understanding of its main antecedent conditions. This study breaks through the limitations of single-factor, linear net-effect research and addresses the shortfall of existing studies that lack exploration from a technological dimension. The innovation of this study lies not in the simple reuse of the TOE framework in a new context, but in leveraging configurational analysis to uncover the interdependent causal structure among the three dimensions—a core pattern that traditional additive regression models struggle to identify. The empirical results reveal three distinct configurational characteristics: first, the driving effect of AI anxiety depends on resource buffers provided by organizations or government support, indicating that the influence of technological factors is contextually conditional rather than universal; second, autonomous decision-making exists only in some high-innovation configurations, serving as a sufficient but not necessary condition, challenging the established assumption in existing research that autonomy is a prerequisite for deviant innovation; third, government support can substitute for missing organizational elements, confirming the existence of cross-dimensional functional complementarity and substitution mechanisms among the three dimensions. These three novel configurational findings—conditionality of factors, non-necessity of elements, and cross-dimensional substitution—expand the traditional use of the TOE framework as merely a tool for dimensional classification, repositioning it as a theoretical analytical framework for explaining multidimensional complex causal relationships. It demonstrates that the multiple triggering pathways of deviant innovative behavior are not simple symmetrical causal relationships, thereby holding significant theoretical expansion value. Specifically, the theoretical contributions are reflected in the following three aspects:

First, it breaks through the limitations of single-factor research and enriches the antecedent network of deviant innovation behavior. Existing research mostly focuses on the independent effects of single dimensions such as individual, organizational, or environmental factors on deviant innovation, while neglecting the interactive effects of multiple concurrent factors. Deviant innovation inherently embodies a dual attribute of “rule-breaking” and “value-creation,” meaning its underlying psychological and motivational dynamics are highly complex and dialectical (Shukla and Kark, 2020). This study integrates six antecedent variables from technological, organizational, and environmental perspectives, demonstrating that employee deviant innovation results from the interactive synergy of multiple factors rather than the linear influence of a single variable, thereby refining the current research framework in this field. This shift from the traditional “net effect” to a “configurational path” analytical perspective further confirms the non-linear characteristics of the emergence of employee innovative behavior in complex system environments (Liu et al., 2026). Concurrently, it provides a more multidimensional psychological explanation for understanding how high-performance work systems and the intrinsic motivation they trigger jointly and systematically drive overall organizational innovation performance (Wang et al., 2024).

Second, it expands the application boundaries of the TOE framework in deviant innovation research and constructs an integrated analytical paradigm for localized deviant innovation. Classic TOE frameworks are rarely used to study employees’ micro-level deviant innovative behavior, which has emerged as a new form of breakthrough innovation in recent years. Furthermore, because its research originated in the West, it has historically been conducted from a Western contextual perspective, lacking localized viewpoints regarding how deep-seated individual orientations or values interact with special transitional institutional environments (Liu and Xu 2022). This study introduces the TOE framework into research on employees’ deviant innovation behavior, incorporates the critical variable of government support in China’s economy, and verifies the cross-level applicability of this framework in studying deviant innovation. As recent cutting-edge research has pointed out, the TOE framework combined with the fsQCA method has strong system-level explanatory power in deconstructing corporate digital technology affordances and the collaborative development capabilities of high-tech industries (Li X. et al., 2025; Zhang et al., 2026; Zhang et al., 2023). The application of this framework in this study not only aligns with this trend but further deepens the configurational path understanding of innovation ecosystem collaboration networks and multi-factor coupling evolution mechanisms from a complex systems perspective (Li et al., 2023; Jiao et al., 2026; Xu et al., 2026).

Third, the study demonstrates that the multiple triggering pathways of deviant innovation behavior are not simple symmetrical causal relationships, deepening the research on causal complexity from a configurational perspective. In modern digitalized or high-pressure workspaces, technology-induced distress and cognitive awareness do not act as simple negative barriers; rather, their interaction with organizational support climates heavily dictates whether employees deploy their psychological resources toward constructive creative deviance or withdrawal. Based on empirical data, this paper identifies three equivalent driving pathways and one inhibiting pathway, successfully revealing the causal asymmetry between high and low levels of deviant innovation behavior. It breaks the traditional thinking limitation of single-variable linear causal research and provides a reference paradigm for studying complex behavioral responses. Furthermore, the findings of this study regarding the complex configurations of the interaction between generative AI competence and technology anxiety are highly consistent with the causal complexity demonstrated in current discussions on how generative AI, as a system element, empowers enterprise product design and business model iterative innovation (Zhang et al., 2026; Xu et al., 2026), providing new empirical support for innovation system theory in the digital era.

### 5.3 Management implications

Employee deviant innovation behavior is not an isolated psychological choice but is deeply influenced by the configuration and interaction of multiple concurrent factors. If enterprises hope to leverage these unconventional behaviors to stimulate overall innovation vitality and achieve leapfrog growth, they must confront the contemporary era of technology anxiety directly, turning passivity into strategic initiative to break management deadlocks. According to the above configurational analysis, management can improve behavioral and organizational interventions in the following specific ways.

#### 5.3.1 Precisely match driving pathways to differentially guide employee innovation behavior

Managers should not use a single Design for the environment and create customized environments for different groups of employees. For employees who are highly anxious about AI and job insecurity but also have a good sense of self-management at work, building a gamified work platform by the company can enhance their feeling of control over their work, and this technological anxiety may be transformed into proactive innovation. On the other hand, for employees who are good at technology but face limited opportunities for expansion at work due to the company’s organization, their managers can introduce relevant policies issued by central or local governments that provide external support for innovation and recognize employees’ extra-curricular projects. For people with average basic abilities, providing them with some introductory Generative AI training and implementing platform-based gamification and other incentives can gradually help them develop a sense of innovation and learn some basic skills. At all times, the managers of differentiated guidance need to be aware that the employees’ trust in AI systems and the corresponding feeling of work autonomy are essential psychological factors for continuous output of innovative behavior in the face of the demands of digital work (Chen and Zhang, 2026). Therefore, the corresponding resource allocation model should aim to build a culture of psychological safety in human-machine collaboration. Various differentiated management interventions need to be dynamically adjusted based on the external organizational environment and individual employee characteristics. In regions supported by policy innovation, the external resource-driven pathway (H1b) holds higher implementation value, allowing managers to fully leverage government policies to convey external support signals to employees. In contrast, organizations in regions lacking institutional resources need to strengthen internal autonomous empowerment and design gamification for motivation, activating the internal configuration pathway (H1a). At the individual level, employees with low AI anxiety are not suitable for using anxiety as a carrier for innovation incentives. Companies can promote positive momentum by enhancing generative AI literacy training and introducing gamified incentives. For employees with high anxiety and low autonomy, psychological reassurance should be implemented first, followed by tiered skill training, and then transitioning to challenging development guidance.

#### 5.3.2 Acknowledge the current era of technological anxiety, turning passivity into active behavioral enablement

Rather than viewing technology-induced stress as a problem to be managed, managers should recognize that such stress is likely unavoidable in the current AI era. It may be difficult to eliminate through strategies, but managers can consider how to provide existing organizational resources to help employees cope with the pressures they currently face. Address the anxiety that some employees may have experienced in light of the rapid development of AI technology and, at the same time, use this upheaval as an opportunity to provide high-end skills training for generative AI. The key is that this approach must be adjusted based on employees’ psychological characteristics. For employees experiencing high AI anxiety and low perceived control, directly emphasizing AI-related threats may backfire, amplifying distress and causing withdrawal behaviors. For such employees, managers should prioritize providing psychological reassurance, clearly communicating issues related to job continuity, and intentionally organizing skill development. Only when employees have sufficient autonomy, skill resources, and institutional buffers of organizational support can anxiety be reappraised as a challenge (Biggs et al., 2017). Thus, it can reduce the specific anxiety that people have felt in the face of artificial intelligence and increase their sense of self-efficacy and cognitive control. At the same time, optimisation of management at both the organizational and environmental levels should be carried out to reduce the perception of risk for employee deviant innovation and provide adequate structural safeguards. In addition, the effectiveness of the above management strategies is highly dependent on the macro-institutional environment. In regions with strong government support for innovation, managers can leverage external policy resources in combination with internal talent development efforts—such as introducing government-sponsored AI skills training programs and innovation R&D subsidies—to strengthen employees perceived institutional support. In areas with relatively limited policy support, enterprise managers need to establish robust internal buffering mechanisms as alternatives, clearly defining the boundaries for trial and error and providing appropriate incentives for the outcomes of such experimentation. This region-specific strategic adjustment helps avoid a one-size-fits-all approach under anxiety-driven management, and by considering the configurational characteristics of internal and external resources that can be mobilized, it enables the customization of suitable management solutions.

#### 5.3.3 Avoid environments that suppress deviant innovation, balancing agile exploration with moderate constraints

Management should be always alert to a low-pressure, low-motivation and low-ability environment that completely suppresses the fundamental drive for employee innovation, such as a low-activation state. Strengthen oversight and impose stringent penalties; at the same time, proactively establish independent off-site zones for inter-departmental cooperation and exploration. Reduce the psychological and material costs of trial-and-error for employees to help managers reduce their anxiety in taking risks and direct their “deviations” toward more productive behaviors that benefit the company.

### 5.4 Research limitations and future directions

Several deficiencies in this study provide reference points for subsequent research in psychology and organizations. The first is that the sample of 326 corporate responses in the empirical study is a cross-sectional one and thus only shows static correlations at a specific time. Given the rapid development of artificial intelligence and changes in organizational policies and external conditions, it is difficult to identify a long-term, dynamic causal chain among these altered reasons. As workplace stress and motivation change constantly over time, future research will be able to collect more all-encompassing, real-time long-term data using the Internet. Second, due to the inherent constraints of the fsQCA method, this study selected only six antecedent conditions, which cannot fully cover the infinite possibilities of triggers underlying deviant innovation behavior. Third, although this study explores deviant innovation within a localized Chinese context across various regions, sizes, and job positions, some sample distribution bias remains, potentially affecting the generalizability of the findings across different enterprise tiers. Because macro-pressures interact uniquely with individual career coping mechanisms, future inquiries should adopt broader theoretical frameworks to fully capture the diverse manifestations and trigger mechanisms of deviant innovation within localized environments.

Finally, although our sample covers different regions, enterprise types, and job levels in China, the research findings are inevitably shaped by China’s specific context, particularly the prominent role of government support in innovation and industrial policies. This unique context enhances the internal validity of our in-depth configuration study within the Chinese setting but limits its direct generalizability to institutional environments with weaker government intervention.

We believe that the configurational logic in the research findings may have cross-contextual generalizability, as it reflects the interactive influence among the three dimensions of technology, organization, and environment in work activities. However, the specific composition of these pathways may vary depending on different institutional contexts. For example, the “externally supported pathway” (H1b) in this study heavily relies on government support as an environmental factor. In contexts with weaker government intervention, this particular pathway may be replaced by other forms of external support. Similarly, the “anxiety-autonomy driven pathway” (H1a) may differ due to culture and social norms that diverge from the current authority.

Therefore, we caution against overgeneralizing contexts where state-enterprise relations or cultural values fundamentally differ. Future cross-national comparative research is needed to assess whether the configurations we identified replicate, diverge, or are replaced in different institutional and cultural environments. Such studies would not only test our findings but also contribute to a more nuanced understanding of how technology, organization, and environmental interactions foster employee deviant innovation behavior across different societies.

## Data Availability

The original contributions presented in the study are included in the article/supplementary material, further inquiries can be directed to the corresponding author.
